# Respiratory omics for early detection of COPD-to-lung cancer transition: mechanisms, biomarkers and clinical translation

**DOI:** 10.3389/fcell.2026.1957266

**Published:** 2026-09-08

**Authors:** Chengyang Liu, Wenhui Jia, Siyuan Jing, Chunling Dong

**Affiliations:** Department of Pulmonary and Critical Care Medicine, Second Hospital, Jilin University, Changchun, China

**Keywords:** COPD, EBC, lung cancer, malignant transformation, respiratory omics, VOCs

## Abstract

Chronic obstructive pulmonary disease (COPD) substantially increases the risk of lung cancer, yet biomarkers capable of identifying malignant transition before radiological abnormalities become evident remain lacking. Respiratory omics has emerged as a promising non-invasive approach for monitoring dynamic molecular alterations during disease progression through the analysis of exhaled volatile organic compounds (VOCs) and exhaled breath condensate (EBC). This review summarizes current evidence on respiratory biomarkers associated with COPD-to-lung cancer transition, emphasizing the biological mechanisms underlying their dynamic evolution and their potential applications in early detection and risk stratification. Rather than considering COPD and lung cancer as separate diseases, we discuss respiratory biomarker changes across the disease continuum and highlight their translational value for identifying high-risk individuals. Finally, we outline the major challenges limiting clinical implementation, including biomarker standardization and prospective validation, and discuss future directions involving multi-omics integration, artificial intelligence, and precision surveillance. Respiratory omics has the potential to advance non-invasive risk assessment and facilitate earlier intervention for COPD-associated lung cancer.

## 1 Introduction

### 1.1 Background and clinical challenges

Chronic obstructive pulmonary disease (COPD) substantially increases the risk of developing lung cancer beyond the effect of smoking alone (Zhang et al., 2025; Zhao et al., 2022). Rather than being entirely separate diseases, COPD and lung cancer are increasingly viewed as different phases of a continuous pathogenic process driven by chronic inflammation, oxidative injury, immune dysfunction, and metabolic alterations. This evolving biological continuum highlights the need for biomarkers capable of identifying early molecular changes associated with malignant progression.

Current lung cancer screening relies predominantly on low-dose computed tomography (LDCT), which is effective for detecting established structural abnormalities but provides limited insight into the biological alterations that precede tumor formation, especially in patients with COPD (Mascalchi and Luconi, 2021; Bonney et al., 2022). This limitation has prompted growing interest in non-invasive biomarkers that can dynamically reflect disease evolution and improve early risk assessment for COPD-associated lung cancer.

### 1.2 The rise of respiratory omics

Respiratory omics has emerged as a promising strategy for monitoring the biological evolution from COPD to lung cancer. By integrating metabolic signatures from exhaled volatile organic compounds (VOCs) with molecular information obtained from exhaled breath condensate (EBC), it enables non-invasive assessment of dynamic alterations within the pulmonary microenvironment (Ratiu et al., 2020; Maniscalco et al., 2024). Compared with conventional imaging, respiratory omics has the potential to detect molecular abnormalities before overt structural changes occur, offering new opportunities for early risk stratification and longitudinal surveillance in high-risk populations (Ibrahim et al., 2021). This narrative review summarizes current evidence on respiratory omics during COPD-to-lung cancer transition, with a particular focus on the dynamic evolution of exhaled biomarkers, their underlying biological mechanisms, and challenges for clinical translation. However, the available evidence remains heterogeneous. Most respiratory biomarkers have been investigated separately in patients with COPD or established lung cancer, whereas direct longitudinal evidence demonstrating their dynamic changes during malignant progression is still limited. Therefore, throughout this review, these biomarkers are interpreted primarily as candidate or indirect markers of COPD-to-lung cancer transition unless supported by longitudinal clinical evidence.

### 1.3 Literature search strategy

A literature search was conducted using PubMed, Web of Science, and Scopus to identify studies relevant to respiratory biomarkers and the progression from COPD to lung cancer. Search terms included combinations of “COPD”, “lung cancer”, “malignant transformation”, “exhaled breath”, “volatile organic compounds”, “VOCs”, “exhaled breath condensate”, “EBC”, “respiratory omics”, and “biomarkers”. Priority was given to peer-reviewed studies published between 2020 and 2026 to reflect recent advances in respiratory omics, analytical technologies, and biomarker discovery. Earlier studies were additionally considered when they provided foundational evidence regarding biomarker discovery, analytical methodology, or biological mechanisms. As this article is a narrative review, studies were selected based on their relevance to COPD, lung cancer, respiratory biomarkers, and potential mechanisms associated with malignant progression.

### 1.4 Pathophysiological basis of the transition from COPD to lung cancer

**FIGURE 1:**
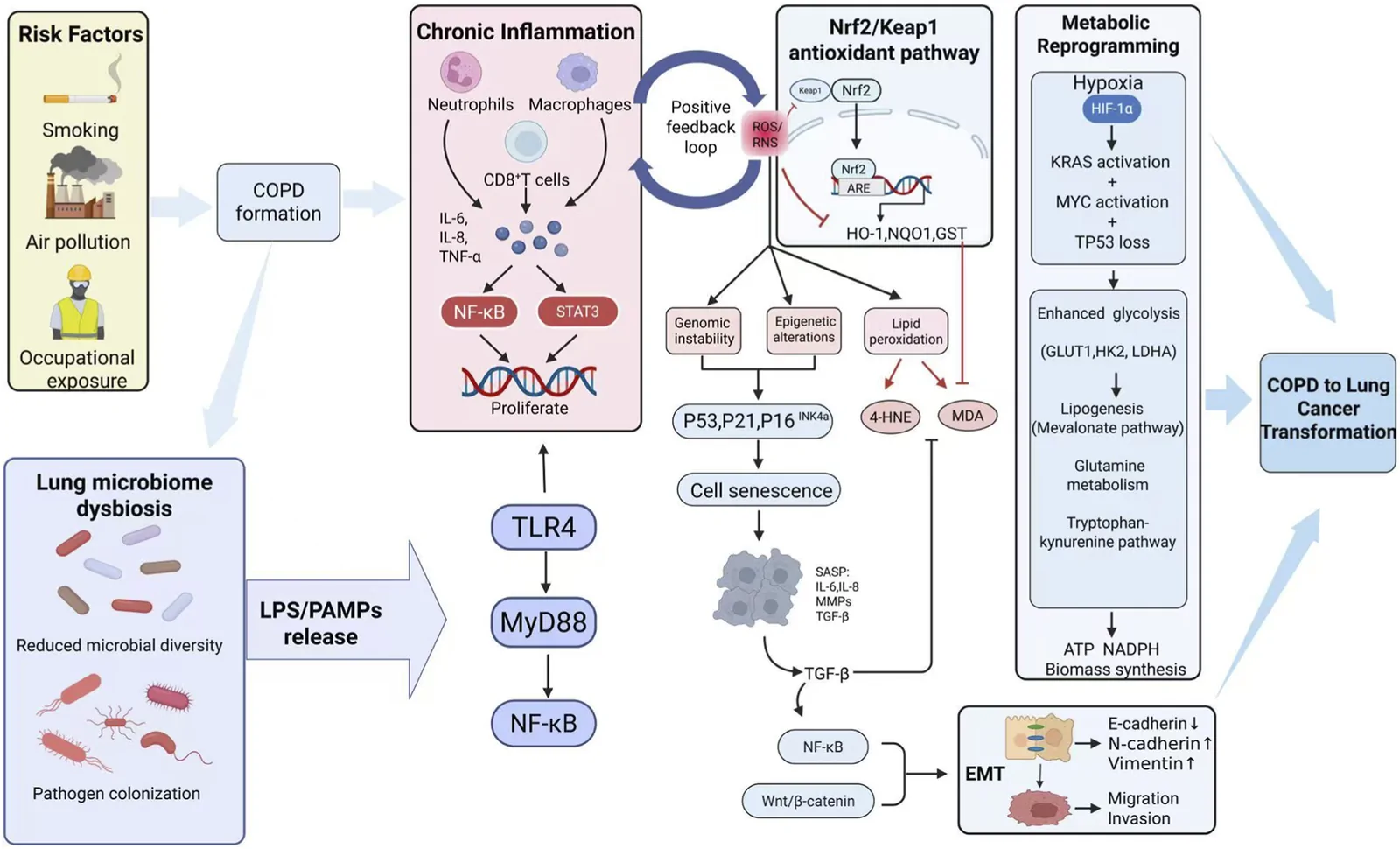
Pathophysiological mechanisms of COPD progression to lung cancer and sources of exhaled breath biomarkers. Chronic exposure to smoking, air pollution, and occupational hazards promotes persistent inflammation, oxidative stress, pulmonary microbiome dysbiosis, and metabolic reprogramming, collectively driving malignant transformation. Dysregulation of NF-κB/STAT3, Nrf2/Keap1, cellular senescence, and epithelial–mesenchymal transition (EMT) contributes to tumor initiation and progression, generating characteristic volatile organic compounds (VOCs) and exhaled breath condensate (EBC) biomarkers. This figure was created with BioRender.

COPD and lung cancer are now widely recognized as interconnected disorders that evolve along a shared pathogenic trajectory rather than as entirely separate diseases. Common environmental exposures, including cigarette smoking, air pollution, and occupational hazards, initiate a series of biological events involving chronic inflammation, oxidative stress, aberrant tissue repair, metabolic rewiring, and pulmonary microbiome dysbiosis, all of which contribute to malignant progression (Figure 1).

Chronic inflammatory activation establishes the biological foundation for this transition. Persistent exposure to inhaled toxic particles promotes the accumulation of neutrophils, macrophages, and CD8^+^ T cells within the airway, resulting in sustained production of TNF-α, IL-6, and IL-8. Beyond maintaining airway injury, these cytokines activate oncogenic signaling pathways, particularly NF-κB and STAT3, thereby supporting epithelial proliferation and immune escape (Wang et al., 2021; Xi et al., 2026). Alterations in the pulmonary microbiome further reinforce this inflammatory state through TLR4/MyD88/NF-κB signaling, creating a microenvironment permissive for tumor development (Wang et al., 2021; Xi et al., 2026).

Oxidative stress acts in parallel with inflammation throughout disease evolution. Excessive generation of reactive oxygen and nitrogen species (ROS/RNS) promotes DNA damage, protein oxidation, lipid peroxidation, and genomic instability (Forder et al., 2023; Caliri et al., 2021). At the same time, disruption of the Nrf2/Keap1 antioxidant pathway weakens cellular defense against oxidative injury, allowing persistent oxidative damage to accumulate and facilitating malignant transformation (Yu and Xiao, 2021; Adinolfi et al., 2023).

Sustained inflammatory and oxidative insults subsequently trigger DNA damage responses and cellular senescence programs, including activation of the p53/p21 and p16/Rb pathways. Senescent cells acquire a senescence associated secretory phenotype (SASP), characterized by continuous release of cytokines, growth factors, and matrix-remodeling enzymes that perpetuate inflammation and reshape the surrounding tissue microenvironment (Zhou et al., 2025; Jha et al., 2024; Brodskaya et al., 2020).

During malignant progression, chronic tissue injury gradually shifts toward phenotypic and metabolic remodeling. Persistent inflammatory signaling induces epithelial–mesenchymal transition (EMT), enabling epithelial cells to acquire migratory and invasive characteristics (Lu et al., 2023). Meanwhile, sustained hypoxia stabilizes HIF-1α and cooperates with EMT-related pathways to remodel the tumor microenvironment (Ye et al., 2024). Concurrent activation of oncogenes such as KRAS and MYC, together with loss of TP53 function, further redirects cellular metabolism toward enhanced glycolysis, lipid synthesis, and amino acid utilization, ultimately supporting tumor growth and malignant evolution (Nong et al., 2020).

## 2 Exhaled breath biomarkers

### 2.1 Characteristic biomarkers in the exhaled breath of patients with COPD

#### 2.1.1 VOCs

Oxidative stress is the predominant driver of volatile organic compound (VOC) alterations in COPD. Persistent accumulation of reactive oxygen and nitrogen species (ROS/RNS) induces lipid peroxidation of membrane polyunsaturated fatty acids, generating characteristic alkanes such as ethane and pentane together with volatile aldehydes including acetaldehyde, hexanal, and heptanal (Ratiu et al., 2020; Tulen et al., 2023). These metabolites reflect ongoing oxidative injury and are considered representative respiratory biomarkers of COPD.

In addition to oxidative stress, chronic hypoxia and metabolic adaptation contribute to altered VOC profiles. Enhanced glycolytic activity and fatty acid β-oxidation increase the production of ketone bodies, particularly acetone, which can be detected in exhaled breath (Xue et al., 2021). Sulfur-containing VOCs, including dimethyl sulfide (DMS) and methyl mercaptan, are also frequently detected and are thought to reflect pulmonary microbiome dysbiosis and persistent bacterial colonization (Wu et al., 2023).

COPD-associated VOCs primarily reflect oxidative stress, metabolic adaptation, and microbial dysbiosis, providing the biological basis for the dynamic evolution of respiratory biomarkers during COPD-to-lung cancer transition (Table 1).

**TABLE 1:** Representative respiratory biomarkers in COPD and lung cancer and their potential relevance to malignant transition.

Biomarker	Sample	COPD evidence	Lung cancer evidence	Representative detection method	Potential relevance to transition	Biological significance	References
Ethane	VOC	Elevated	Elevated	GC based breath analysis, including GC–MS	Indirect candidate marker	Lipid peroxidation	(Ratiu et al., 2020; Tulen et al., 2023; Matsuoka et al., 2025)
Pentane	VOC	Elevated	Elevated	GC based breath analysis, including GC–MS	Indirect candidate marker	Oxidative stress-induced membrane damage	(Ratiu et al., 2020; Tulen et al., 2023; Matsuoka et al., 2025)
Hexanal	VOC	Elevated	Markedly elevated	GC based breath analysis, including GC–MS	Promising candidate marker	ω-6 PUFA oxidation and lipid remodeling	(Tulen et al., 2023; Lundberg et al., 2025; Matsuoka et al., 2025)
Nonanal	VOC	Rarely reported	Elevated	GC based breath analysis, including GC–MS	Exploratory candidate marker	Membrane remodeling and tumor proliferation	(Lundberg et al., 2025; Matsuoka et al., 2025)
Decanal	VOC	Rarely reported	Elevated	GC based breath analysis, including GC–MS	Exploratory candidate marker	Enhanced membrane synthesis and tumor growth	(Lundberg et al., 2025; Matsuoka et al., 2025)
Acetone	VOC	Elevated	Markedly elevated	GC–MS, GC–IMS or direct MS breath analysis	Indirect candidate marker	Enhanced glycolysis (Warburg effect)	(Xue et al., 2021; Lundberg et al., 2025; Matsuoka et al., 2025)
Isoprene	VOC	Elevated	Elevated	GC–MS,PTR-MS or related real-time MS techniques	Supportive metabolic marker	Cholesterol biosynthesis and cell proliferation	(Mochalski et al., 2024)
Dimethyl sulfide	VOC	Elevated	Elevated	GC–MS or sulfur-sensitive GC analysis	Indirect candidate marker	Microbiome dysbiosis and sulfur amino acid metabolism	(Wu et al., 2023)
Phenylacetaldehyde	VOC	Occasionally reported	Elevated	GC based breath analysis, including GC–MS	Exploratory candidate marker	Aromatic amino acid metabolic reprogramming	(Wang D et al., 2024)
IL-6	EBC	Elevated	Elevated	ELISA or multiplex cytokine assay	Supportive inflammatory marker	Persistent inflammatory activation	(Szucs et al., 2019; Shyam et al., 2021; Uliński et al., 2022)
IL-8	EBC	Elevated	Elevated	ELISA or multiplex cytokine assay	Supportive inflammatory marker	Neutrophil recruitment and chronic inflammation	(Szucs et al., 2019; Shyam et al., 2021; Uliński et al., 2022)
TNF-α	EBC	Elevated	Elevated	ELISA or multiplex cytokine assay	Supportive inflammatory marker	NF-κB activation and inflammatory amplification	(Szucs et al., 2019; Shyam et al., 2021; Uliński et al., 2022)
H_2_O_2_	EBC	Elevated	Markedly elevated	Spectrophotometric, fluorometric or enzymatic assays	Supportive oxidative-stress marker	ROS accumulation and oxidative damage	(Barnes, 2020; Taniguchi et al., 2021; Freund et al., 2024; Dos Santos et al., 2022; Kolbasina et al., 2020)
8-Isoprostane	EBC	Elevated	Markedly elevated	Enzyme immunoassay or chromatographic analysis	Supportive oxidative-stress marker	Lipid peroxidation	(Barnes, 2020; Taniguchi et al., 2021; Freund et al., 2024; Dos Santos et al., 2022; Kolbasina et al., 2020)
MDA	EBC	Elevated	Elevated	Chromatographic or colorimetric lipid-peroxidation assays	Supportive oxidative-stress marker	Oxidative membrane injury	(Barnes, 2020; Taniguchi et al., 2021; Freund et al., 2024; Dos Santos et al., 2022; Kolbasina et al., 2020; Matsuoka et al., 2025)
MMP-9	EBC	Elevated	Elevated	ELISA or multiplex cytokine assay	Exploratory candidate marker	Extracellular matrix degradation and EMT	(Dimic-Janjic et al., 2023; Uliński et al., 2022; Poto et al., 2022)
VEGF	EBC	Elevated	Elevated	ELISA or multiplex cytokine assay	Supportive angiogenic marker	Angiogenesis and tumor microenvironment remodeling	(Chis et al., 2021; Poto et al., 2022; Wiegman et al., 2020; Nicola et al., 2021; Qin et al., 2021; He et al., 2024)
miR-21	EBC	Occasionally reported	Elevated	qRT-PCR and miRNA profiling/sequencing	Exploratory candidate marker	Oncogenic signaling and proliferation	(Pérez-Sánchez et al., 2021; Rai et al., 2023; Zanoaga et al., 2019; Ferrari et al., 2025)
miR-155	EBC	Occasionally reported	Elevated	qRT-PCR and miRNA profiling/sequencing	Exploratory candidate marker	Inflammation-associated carcinogenesis	(Pérez-Sánchez et al., 2021; Rai et al., 2023; Zanoaga et al., 2019; Ferrari et al., 2025)
ctDNA	EBC	Not reported	Detectable	Targeted PCR, ultrasensitive PCR and NGS based mutation analysis	Exploratory candidate marker	Genomic instability and driver mutation detection	(Koc et al., 2019; Ryan et al., 2022)

#### 2.1.2 EBC

Compared with VOCs, exhaled breath condensate (EBC) more directly reflects inflammatory and molecular alterations within the airway microenvironment. Elevated levels of inflammatory mediators, including IL-6, IL-8, TNF-α, and leukotriene B4 (LTB4), are consistently observed in patients with COPD and correlate with disease severity (Szucs et al., 2019; Shyam et al., 2021). Oxidative stress biomarkers, particularly hydrogen peroxide (H_2_O_2_), 8-isoprostane, and malondialdehyde (MDA), are also increased, reflecting persistent oxidative injury and lipid peroxidation (Barnes, 2020; Taniguchi et al., 2021; Freund et al., 2024; Dos Santos et al., 2022).

In addition, increased concentrations of cell-free DNA (cfDNA), mitochondrial DNA (mtDNA), and matrix metalloproteinases especially MMP-9 and MMP-12 indicate ongoing cellular injury and extracellular matrix remodeling in COPD (Kazeminasab et al., 2020; Dimic-Janjic et al., 2023). Angiogenesis-related factors, such as vascular endothelial growth factor (VEGF), have likewise been associated with hypoxia-induced vascular remodeling and disease progression (Chis et al., 2021). Collectively, these EBC biomarkers characterize the inflammatory and oxidative microenvironment of COPD and provide a molecular basis for monitoring subsequent malignant transformation (Table 1).

### 2.2 Characteristic biomarkers in the exhaled breath of patients with lung cancer

#### 2.2.1 VOCs

Compared with COPD, the VOC profile of lung cancer more prominently reflects metabolic reprogramming associated with malignant progression (Qi et al., 2022; Yan et al., 2024). Enhanced lipid peroxidation and membrane remodeling contribute to increased levels of long-chain aldehydes, including hexanal, heptanal, nonanal, decanal, and 2-pentenal, which are considered characteristic metabolites of tumor-associated oxidative stress (Lundberg et al., 2025).

Lung cancer is also characterized by profound metabolic alterations. Enhanced glycolysis promotes the accumulation of volatile ketones and alcohols, such as acetone, 2-butanone, ethanol, and n-propanol (Lundberg et al., 2025; Chu et al., 2024), while activation of the mevalonate pathway contributes to increased isoprene production, reflecting accelerated cholesterol biosynthesis and cell proliferation (Mochalski et al., 2024). In addition, altered cytochrome P450 activity influences the metabolism of aromatic hydrocarbons, including benzene, toluene, p-xylene, and styrene, further contributing to the characteristic VOC signature of lung cancer (Huang et al., 2023; Abu-Bakar et al., 2022).

Lung cancer associated VOCs reflect tumor-related metabolic reprogramming and provide an important reference for identifying dynamic biomarker changes during COPD-to-lung cancer transition (Table 1).

#### 2.2.2 EBC

Exhaled breath condensate (EBC) provides direct access to molecular alterations associated with lung cancer. Among the most extensively investigated biomarkers are nucleic acids, particularly oncogenic microRNAs. Increased expression of miR-21, miR-155, and diagnostic miRNA panels comprising miR-4507, miR-6777–5p, and miR-451a has demonstrated promising potential for early lung cancer detection (Pérez-Sánchez et al., 2021; Rai et al., 2023; Zanoaga et al., 2019; Ferrari et al., 2025). Long non-coding RNAs (lncRNAs) have also emerged as candidate biomarkers owing to their roles in tumor progression and epithelial–mesenchymal transition (Tetik Vardarlı et al., 2023).

Tumor-derived nucleic acids, especially circulating tumor DNA (ctDNA), can also be detected in EBC. High-sensitivity assays have successfully identified mutations in major driver genes, including EGFR, KRAS, ALK, and TP53, highlighting the potential of EBC as a non-invasive liquid biopsy platform (Koc et al., 2019; Ryan et al., 2022).

In addition to nucleic acids, oxidative stress markers such as H_2_O_2_ and MDA remain elevated in patients with lung cancer, while protein biomarkers including carcinoembryonic antigen (CEA) have shown potential for rapid respiratory biomarker detection (Kolbasina et al., 2020; Zou et al., 2020). Collectively, these EBC biomarkers reflect multiple aspects of tumor biology and provide a molecular basis for monitoring COPD-to-lung cancer transition (Table 1).

## 3 Research into potential molecular biomarkers of transformation and their mechanisms

### 3.1 Transformation characteristics of VOCs

Based on currently available cross-sectional and mechanistic evidence, the VOC profile may undergo a progressive shift during COPD to lung cancer transition characterized by a shift from being primarily driven by oxidative stress to being driven by multiple metabolic reprogramming processes. VOCs during the COPD stage primarily reflect chronic inflammation and persistent oxidative damage, with lipid peroxidation related products predominating; however, as malignant transformation occurs, the lung cancer stage gradually exhibits a variety of metabolic features, including lipid metabolic reorganisation, enhanced glycolysis, abnormal amino acid metabolism and changes in the microenvironment, forming a more complex respiratory metabolic fingerprint. Consequently, the dynamic changes in the VOC profile not only reflect the transition of lung tissue from a state of chronic damage to one of malignant proliferation, but also dynamic changes in VOC profiles may reflect the transition of lung tissue from chronic injury to malignant proliferation and provide a biological basis for identifying key molecular events and potential early-warning biomarkers during COPD-to-lung cancer progression. The following sections will elaborate on these aspects in terms of lipid peroxidation, glycolytic metabolism and amino acid metabolism (Table 2).

**TABLE 2:** Respiratory biomarkers for early warning of malignant transformation from COPD to lung cancer.

Mechanism	Biomarker	Sample type	Change trend	Clinical implication	References
Chronic inflammation	IL-6	EBC	↑	Persistent inflammatory activation	(Szucs et al., 2019; Shyam et al., 2021; Uliński et al., 2022)
	IL-8	EBC	↑	Neutrophil recruitment	(Szucs et al., 2019; Shyam et al., 2021; Uliński et al., 2022)
	TNF-α	EBC	↑	NF-κB activation	(Szucs et al., 2019; Shyam et al., 2021; Uliński et al., 2022)
Oxidative stress	H_2_O_2_	EBC	↑↑	ROS accumulation	(Barnes, 2020; Taniguchi et al., 2021; Freund et al., 2024; Dos Santos et al., 2022; Kolbasina et al., 2020)
	8-Isoprostane	EBC	↑↑	Lipid peroxidation	(Barnes, 2020; Taniguchi et al., 2021; Freund et al., 2024; Dos Santos et al., 2022; Kolbasina et al., 2020)
	MDA	EBC	↑↑	Oxidative membrane damage	(Barnes, 2020; Taniguchi et al., 2021; Freund et al., 2024; Dos Santos et al., 2022; Kolbasina et al., 2020; Matsuoka et al., 2025)
Lipid remodeling	4-HNE	EBC	↑↑	Lipid peroxidation and pro-carcinogenic signaling	(Matsuoka et al., 2025; Fakhrioliaei et al., 2024; Milkovic et al., 2023; Fratta Pasini et al., 2020; Li et al., 2020)
	Hexanal	VOC	↑↑	ω-6 PUFA oxidation and membrane turnover	(Tulen et al., 2023; Lundberg et al., 2025; Matsuoka et al., 2025; Milkovic et al., 2023; Fratta Pasini et al., 2020)
	Nonanal	VOC	↑↑	Tumor-associated lipid metabolism	(Lundberg et al., 2025; Matsuoka et al., 2025; Milkovic et al., 2023; Fratta Pasini et al., 2020)
Glycolytic reprogramming	Acetone	VOC	↑↑	Warburg effect and altered energy metabolism	(Xue et al., 2021; Lundberg et al., 2025; Chu et al., 2024; Tang et al., 2024; Furuhashi et al., 2024; Abukwaik et al., 2024; Fu et al., 2022; Yang et al., 2024)
Amino acid metabolism	Phenylacetaldehyde	VOC	↑↑	Amino acid metabolic dysregulation	(Wang D. et al., 2024)
Microbial dysbiosis/sulfur metabolism	Dimethyl sulfide	VOC	↑↑	Sulfur metabolism and microbiome alteration	(Wu et al., 2023)
EMT	MMP-9	EBC	↑↑	Extracellular matrix degradation	(Dimic-Janjic et al., 2023; Uliński et al., 2022; Poto et al., 2022)
	TIMP-1	EBC	↓	MMP/TIMP imbalance and abnormal repair	(Dimic-Janjic et al., 2023; Uliński et al., 2022)
Angiogenesis	VEGF	EBC	↑↑	Hypoxia-related angiogenesis and tumor microenvironment remodeling	(Chis et al., 2021; Poto et al., 2022; Wiegman et al., 2020; Nicola et al., 2021; Zheng and Jin, 2025)
	Ang-2	EBC	↑	Angiogenic switch and vascular remodeling	(Qin et al., 2021; He et al., 2024)
Nucleic acid release	cfDNA	EBC	↑	Cell injury and genomic instability	(Kazeminasab et al., 2020; Koc et al., 2019)
	mtDNA	EBC	↑	Oxidative stress and innate immune activation	(Kazeminasab et al., 2020; Kolbasina et al., 2020)
​	miR-21	EBC	↑↑↑	Oncogenic activation	(Pérez-Sánchez et al., 2021; Rai et al., 2023; Zanoaga et al., 2019; Ferrari et al., 2025)
​	miR-155	EBC	↑↑	Inflammation-driven carcinogenesis	(Pérez-Sánchez et al., 2021; Rai et al., 2023; Zanoaga et al., 2019; Ferrari et al., 2025)
​	ctDNA	EBC	↑↑↑	Early malignant transformation and driver mutation detection	(Koc et al., 2019; Ryan et al., 2022)

#### 3.1.1 Lipid metabolic reprogramming

COPD patients are subjected to chronic hypoxia and persistent inflammation over the long term; this unique microenvironment is considered a key pathological basis driving the malignant transformation of lung tissue. Hypoxia promotes the stable expression of HIF-1α, which in turn activates the cyclooxygenase-2 (COX-2) and its downstream prostaglandin E2 (PGE2) signaling pathways (Finetti et al., 2020). PGE2 not only promotes cell proliferation and angiogenesis but also induces the polarisation of tumor-associated macrophages towards the M2 phenotype, thereby creating an immunosuppressive microenvironment that facilitates malignant transformation (Gómez-Valenzuela et al., 2021; Zheng et al., 2025). Persistent hypoxia and an inflammatory microenvironment lead to the continuous accumulation of ROS, which not only contribute to the formation of the PGE2-mediated pro-cancerous microenvironment but also act as key upstream drivers of lipid peroxidation.

**FIGURE 2:**
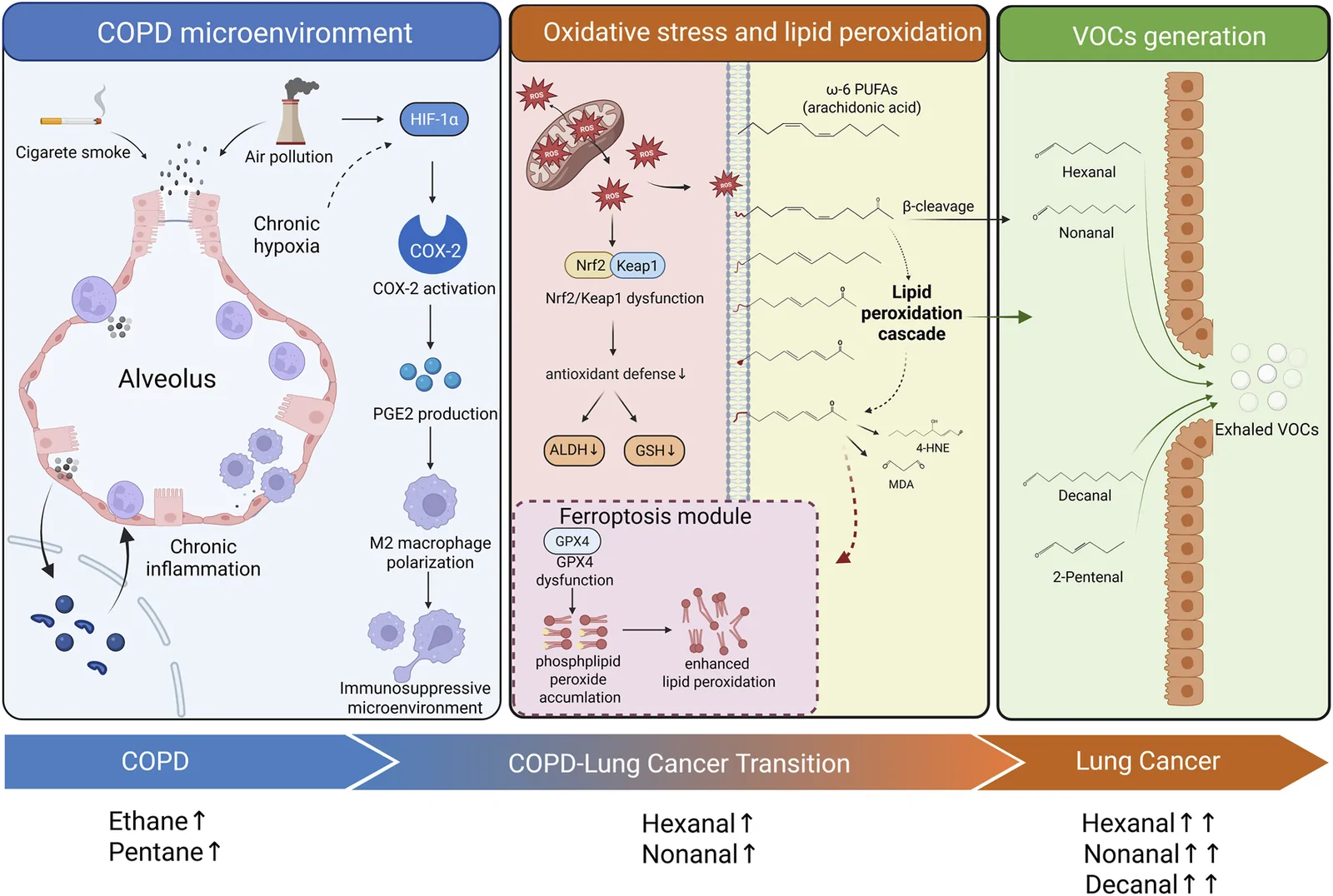
Evolution of VOCs during lipid metabolic reprogramming in COPD-to-lung cancer transition. Chronic hypoxia and oxidative stress induce lipid peroxidation through HIF-1α/COX-2/PGE2 signaling, Nrf2/Keap1 dysregulation, and ferroptosis-related pathways. Consequently, exhaled VOCs shift from short-chain alkanes in COPD to tumor-associated aldehydes, including hexanal, nonanal, and decanal, during lung cancer development. This figure was created with BioRender.

Lipid peroxidation is a key molecular event linking chronic damage in COPD to the development of lung cancer. Under normal conditions, PUFAs in cell membranes maintain homeostasis under the protection of the antioxidant system; however, under sustained ROS attack, membrane structures rich in ω-6 polyunsaturated fatty acids are particularly susceptible to peroxidation reactions (Lee et al., 2021). As lung cancer develops, tumor cells continuously synthesise new cell membranes to meet the demands of rapid proliferation, leading to a further increase in the levels of ω-6 PUFAs such as arachidonic acid, thereby enhancing the pool of substrates for lipid peroxidation. Studies have shown that ω-6 PUFAs, via radical-mediated chain reactions, can produce reactive lipid peroxidation compounds that include MDA and 4-hydroxynonenal (4-HNE), which are further cleaved to form hexanal, nonanal, decanal and 2-pentenal, among other volatile aldehydes (Matsuoka et al., 2025). These aldehydes not only diffuse into exhaled breath but also form adducts with DNA and proteins, promoting genetic mutations and abnormal cell signaling. In this process, the Nrf2/Keap1 antioxidant defence system plays a key regulatory role. Nrf2 can induce the expression of genes related to aldehyde dehydrogenase (ALDH), glutathione peroxidase (GPX) and glutathione (GSH) synthesis, thereby clearing lipid peroxidation products and maintaining redox homeostasis (Fakhrioliaei et al., 2024; Milkovic et al., 2023). However, during prolonged oxidative stress and tumor progression, an imbalance in the Nrf2/Keap1 pathway can lead to a decline in aldehyde clearance capacity, resulting in the persistent accumulation of end-products such as hexanal, nonanal and decanal in lung tissue and their release into exhaled breath (Milkovic et al., 2023; Fratta Pasini et al., 2020). Emerging evidence suggests that loss of GPX4 activity and the subsequent enhancement of ferroptosis-associated lipid peroxidation facilitate the accumulation of oxidized membrane phospholipids, ultimately leading to increased generation of reactive aldehydes, including 4-HNE and MDA (Li et al., 2020). As lipid peroxidation is a major source of volatile aldehydes, ferroptosis-related metabolic abnormalities may influence the composition of the VOC profile during the lung cancer stage; but further research is needed to fully understand the precise pathways. In addition to long-chain aldehydes, short-chain alkanes such as ethane and pentane are also important volatile products of lipid peroxidation; their levels are already elevated in the COPD stage and increase further as oxidative damage persists. By contrast, long-chain aldehydes such as hexanal, nonanal and decanal are better indicators of lipid remodeling in tumor cell membranes and abnormal proliferation; they are therefore considered important candidate biomarkers for distinguishing between simple COPD and the transition stage to lung cancer. Furthermore, elevated levels of isoprene may also reflect activation of the mevalonate pathway and enhanced cell membrane biosynthesis, which are closely associated with tumor progression (Alfalasi et al., 2024; Wang et al., 2023) (Figure 2).

In summary, hypoxia-induced lipid peroxidation permeates the entire process of COPD-to-lung cancer transformation, with the VOC profile driven by this process gradually shifting from oxidative damage markers represented by ethane and pentane to tumor-associated metabolites represented by long-chain aldehydes such as hexanal, nonanal and decanal. This process not only reflects a continuous increase in the severity of oxidative stress but also illustrates the evolution of lung tissue from a state of oxidative damage driven by chronic inflammation to a state of tumor-associated metabolic reprogramming.

#### 3.1.2 Glycolytic metabolic reprogramming

Glycolytic metabolic reprogramming is one of the most representative metabolic events in the malignant transformation of COPD into lung cancer; its essence is manifested in the gradual evolution of cellular energy acquisition from hypoxia-adaptive glycolysis to the sustained Warburg metabolism characteristic of tumor cells. During the COPD stage, long-term airflow limitation and destruction of alveolar structure lead to localised chronic hypoxia. HIF-1α is expressed at a consistently stable level and, by transcriptionally activating key enzymes such as GLUT1, HK2, PFK and LDHA, promotes glucose uptake and enhances glycolysis (Zheng and Jin, 2025). At this stage, the upregulation of glycolysis primarily constitutes an adaptive compensatory mechanism, aimed at maintaining the cell’s energy supply and viability in a hypoxic environment. As inflammation persists, lactic acid accumulates in large quantities and induces local acidification, further promoting the recruitment of inflammatory cells, angiogenesis and immune regulation, thereby providing a metabolic foundation for subsequent malignant transformation.

As lung tissue progresses toward a precancerous state and subsequent malignant transformation, glycolytic enhancement becomes increasingly independent of HIF-1α-mediated hypoxic responses. Instead, persistent inflammation and oxidative stress promote the activation of oncogenic signaling networks, including NF-κB, STAT3, and PI3K/Akt/mTOR pathways. These molecular events facilitate KRAS and MYC dysregulation together with functional impairment of TP53, ultimately driving sustained metabolic reprogramming toward a Warburg-like glycolytic phenotype (Tang et al., 2024; Furuhashi et al., 2024). Loss of TP53 function lifts its inhibitory effect on glycolysis, whilst simultaneously reducing the metabolic constraints mediated by TIGAR, causing glucose metabolic flux to persistently favour glycolysis and the pentose phosphate pathway (Liao et al., 2025; Abukwaik et al., 2024; Fu et al., 2022). Even under conditions of adequate oxygen supply, tumor cells still prioritise glycolysis for energy production, thereby exhibiting the classic Warburg effect. Concurrently, the pentose phosphate pathway is activated, producing high quantities of ribose-5-phosphate and NADPH, which serve as raw materials for the synthesis of proteins, lipids, and nucleic acids, therefore promoting the fast growth of tumor cells (Ratiu et al., 2020).

**FIGURE 3:**
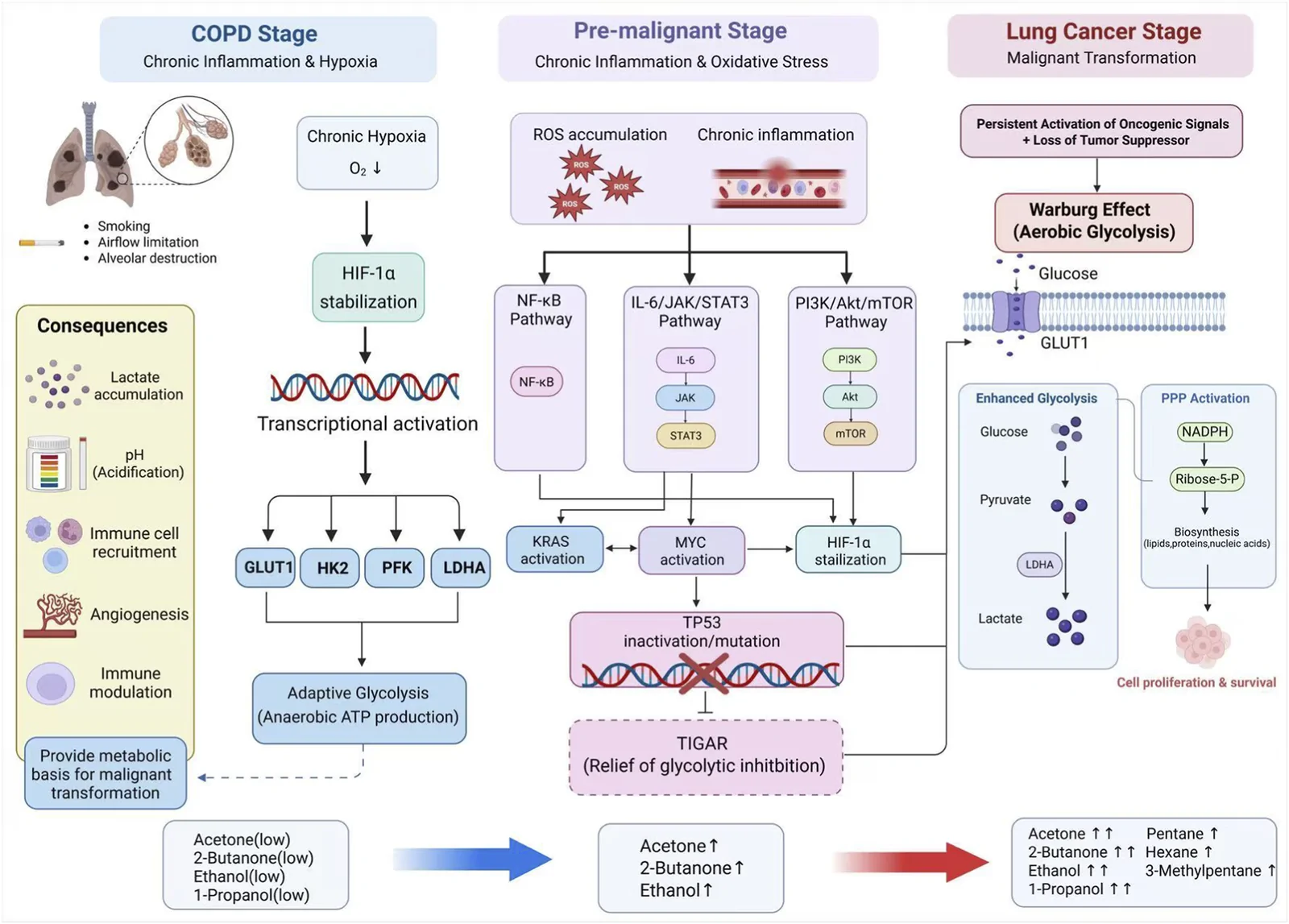
Evolution of exhaled breath biomarkers during glycolytic metabolic reprogramming in COPD-to-lung cancer transition. The transition from hypoxia-driven adaptive glycolysis in COPD to oncogene-mediated Warburg metabolism in lung cancer promotes enhanced biosynthesis and the accumulation of characteristic exhaled metabolites. Ketones and alcohols, including acetone, 2-butanone, ethanol, and n-propanol, may therefore serve as potential biomarkers of malignant transformation. This figure was created with BioRender.

Increased glycolytic flux and the resulting downstream metabolic reprogramming may further influence the composition of VOCs in exhaled breath. Levels of ketones and alcohols, such as acetone, 2-butanone, ethanol and n-propanol, are significantly elevated in the exhaled breath of lung cancer patients. These metabolites are, on the one hand, associated with changes in pyruvate metabolism following enhanced glycolysis and, on the other hand, closely related to fatty acid oxidation, ketone body metabolism and redox imbalance (Lundberg et al., 2025; Fakhrioliaei et al., 2024; Yang et al., 2024). Furthermore, the large amounts of NADPH generated under conditions of high glycolysis not only support cellular biosynthetic demands but also promote lipid metabolic reorganisation and lipid peroxidation, thereby indirectly increasing the production of volatile alkanes such as pentane, hexane and 3-methylpentane (Figure 3).

Both COPD and lung cancer are characterized by enhanced glycolysis and elevated levels of associated VOCs; however, there are marked differences between the two conditions in terms of severity and metabolic drivers. The COPD stage is primarily characterized by HIF-1α-mediated reversible compensatory responses, whereas the lung cancer stage involves persistent metabolic reprogramming driven by genetic and epigenetic alterations. Consequently, the dynamic changes in ketones and alcohols—such as acetone, 2-butanone and ethanol—in conjunction with VOCs associated with lipid peroxidation, may serve as important metabolic signals reflecting the malignant transformation from COPD to lung cancer, providing potential biomarkers for the development of early-warning models based on exhaled breath.

#### 3.1.3 Amino acid metabolic reprogramming

Amino acid metabolic reprogramming plays a key role in shaping the tumor microenvironment, regulating the immune response and maintaining redox homeostasis; its metabolites can also reflect the evolutionary process of lung tissue transitioning from chronic inflammation to malignancy via exhaled VOCs (Wang D. et al., 2024). During the COPD stage, persistent chronic inflammation and oxidative stress can lead to a gradual imbalance in the pulmonary immune microenvironment. Prolonged inflammatory stimulation induces macrophages, dendritic cells and other immune cells to express indole-2,3-dioxygenase (IDO1), thereby promoting the activation of the tryptophan-kynurenine (Trp-Kyn) metabolic pathway (Akhmetova et al., 2022). As the disease progresses and a precancerous microenvironment develops, the expression of IDO1 and tryptophan 2,3-dioxygenase (TDO) increases further, resulting in the breakdown of large amounts of tryptophan into kynurenine (Kyn). On the one hand, tryptophan depletion limits the proliferation of effector T cells; on the other hand, kynurenine, as an aryl hydrocarbon receptor (AhR) ligand, promotes the differentiation of regulatory Tregs and suppresses CD8^+^ T cell function, thereby creating an immunosuppressive tumor microenvironment (Akhmetova et al., 2022; Basson et al., 2023; Zhang et al., 2024; Pamart et al., 2024). This suggests that the sustained activation of the Trp-Kyn-AhR axis is one of the key mechanisms linking chronic inflammation to immune evasion in lung cancer.

Abnormal tryptophan metabolism can also disrupt the aromatic amino acid metabolic network. Under conditions of sustained oxidative stress, the oxidative inactivation of tetrahydrobiopterin (BH_4_) increases, which can affect the activity of phenylalanine hydroxylase (PAH), thereby limiting the conversion of phenylalanine to tyrosine. Imbalances in aromatic amino acid metabolism not only affect the synthesis of neurotransmitters and cellular signaling molecules, but may also promote the formation of aromatic volatile organic compounds (VOCs) such as phenethylaldehyde (Zeitler and Murray, 2023). In recent years, several exhaled breath omics studies have found that levels of aromatic VOCs, such as phenethylaldehyde, are significantly elevated in lung cancer patients, suggesting that they may reflect tumor-associated abnormalities in amino acid metabolism.

**FIGURE 4:**
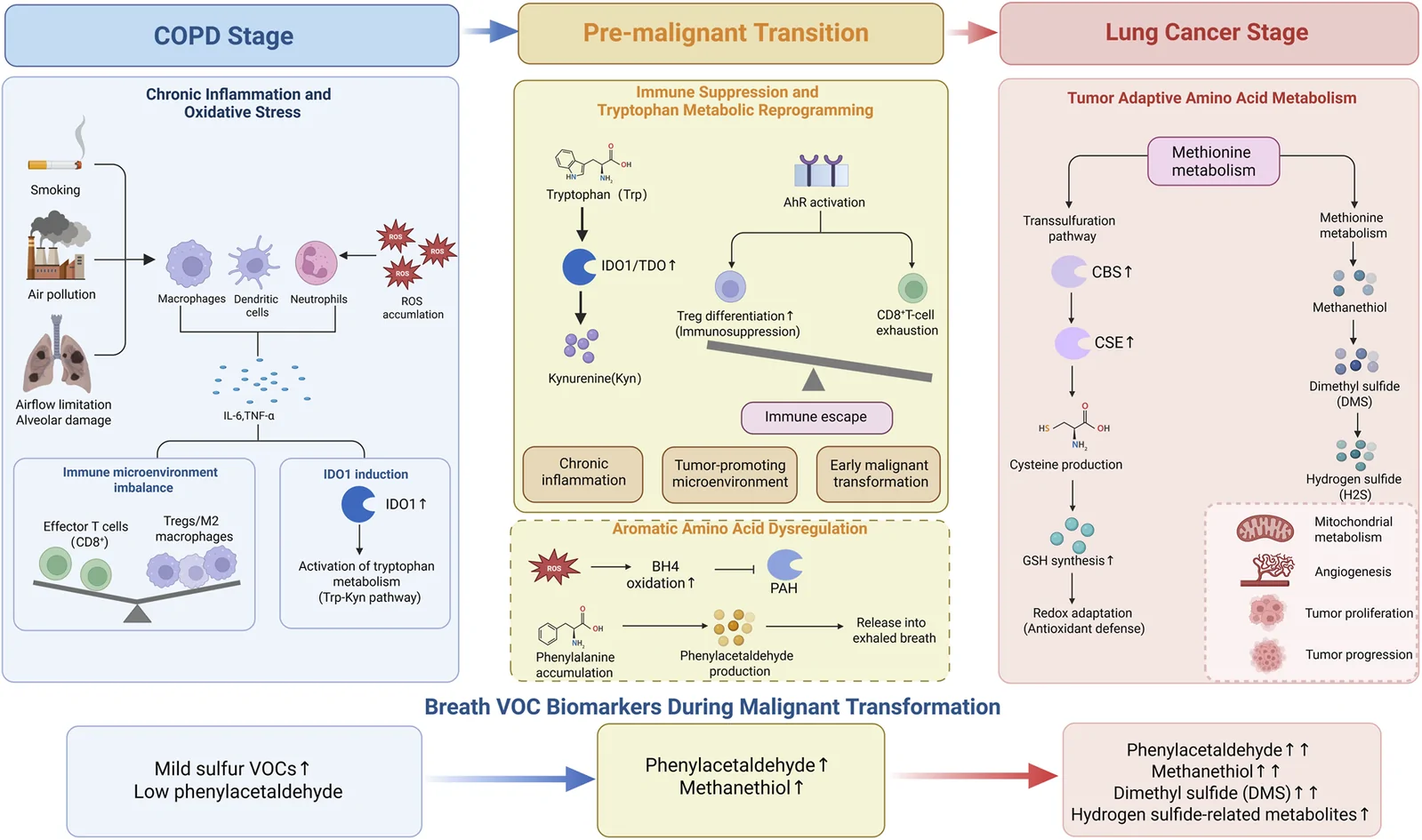
Evolution of VOCs during amino acid metabolic reprogramming in COPD-to-lung cancer transition. Amino acid metabolic reprogramming, including activation of the Trp–Kyn pathway and alterations in aromatic and sulphur amino acid metabolism, contributes to immune evasion and adaptive tumor growth during malignant transformation. Correspondingly, characteristic VOCs, such as phenethylaldehyde, methanethiol, and dimethyl sulphide (DMS), progressively accumulate and may provide early warning signals for lung carcinogenesis in patients with COPD. This figure was created with BioRender.

In addition to aromatic amino acids, the metabolic reprogramming of sulphur-containing amino acids also runs throughout from COPD to lung cancer. During the COPD stage, in response to persistent oxidative damage, the body increases cystine production by activating the transsulfation pathway to maintain glutathione (GSH) synthesis and antioxidant capacity (Zhu et al., 2019). During this process, the expression of cystathionine β-synthase (CBS) and cystathionine γ-lyase (CSE) is upregulated, promoting the conversion of homocysteine to cysteine (Zhu et al., 2019). As tumors develop, the demand for methionine and glutathione in cancer cells increases further; sulphur-containing amino acid metabolism remains highly active, leading to the production of volatile sulphur-containing compounds such as methanethiol, dimethyl sulphide (DMS) and hydrogen sulphide (H_2_S) (Philipp et al., 2023). These sulfur-containing metabolites may not only reflect changes in redox status but also participate in the regulation of mitochondrial metabolism, angiogenesis, and tumor cell proliferation (Figure 4).

Consequently, from the activation of sulphur-transfer pathways—predominantly characterized by antioxidant compensation during the COPD stage—to the reprogramming of amino acid metabolism—characterized by immune evasion and adaptive tumor growth during the lung cancer stage—the Trp-Kyn-AhR axis, abnormalities in aromatic amino acid metabolism, and the remodeling of sulphur-containing amino acid metabolism collectively drive the formation of characteristic VOC spectra. Among these, volatile metabolites such as phenethylaldehyde, methyl mercaptan and dimethyl sulphide show promise as important exhaled breath biomarkers reflecting the malignant transformation from COPD to lung cancer (Philipp et al., 2023).

### 3.2 Metabolic characteristics in EBC

Whereas VOCs primarily reflect changes in pulmonary metabolic end-products, exhaled breath condensate (EBC) can more directly reflect inflammatory responses, oxidative damage, vascular remodeling and abnormal gene expression in the airway microenvironment; it is therefore considered an important non-invasive diagnostic tool for studying the malignant transformation of COPD into lung cancer. As the disease progresses, molecular biomarkers in EBC exhibit dynamic changes, evolving gradually from chronic inflammation and oxidative stress towards enhanced angiogenesis, genomic instability and the activation of tumor-associated signaling pathways. For instance, oxidative stress and lipid peroxidation-related products such as 8-isoprostane, 4-HNE and MDA are abnormally elevated in both COPD and lung cancer patients, suggesting that persistent oxidative damage may be involved in the malignant transformation process. Concurrently, the abnormal expression of angiogenesis-related factors such as VEGF and Ang-2 reflects a shift in the pulmonary microenvironment from a state of chronic injury to one that promotes tumor angiogenesis. Furthermore, various miRNAs and other nucleic acid molecules can be reliably detected in EBC; some of these lung cancer-associated miRNAs have already demonstrated good diagnostic value, suggesting they hold promise as possible biomarkers to track the risk of malignant transformation in COPD(Table 2).

#### 3.2.1 Markers associated with inflammation, oxidative stress and tissue damage

Chronic exposure to cigarette smoke and other inhaled particulates sustains the activity of inflammatory pathways, including NF-κB and AP-1, within airway epithelial cells and alveolar macrophages. Persistent activation of these signaling networks promotes the continuous production of cytokines such as IL-6, IL-8, and TNF-α, thereby establishing a pro-inflammatory pulmonary microenvironment that favours disease progression (Uliński et al., 2022). Concurrently, an imbalance in the expression of matrix metalloproteinase-9 (MMP-9) and its tissue inhibitor (TIMP-1) leads to persistent degradation and abnormal repair of the extracellular matrix; changes in their ratio are considered key molecular hallmarks of pulmonary tissue remodeling and the formation of a precancerous microenvironment (Dimic-Janjic et al., 2023; Wang Y. et al., 2024).

In addition to inflammatory factors, changes in markers associated with oxidative damage in EBC can also reflect the progression of the disease from chronic injury to malignant transformation. Prolonged inflammatory stimulation and excessive accumulation of ROS can induce lipid peroxidation, producing stable products such as 8-isoprostane,4-HNE and MDA. Among the oxidative stress indicators identified in EBC, 8-isoprostane is widely regarded as a robust *in vivo* marker of lipid peroxidation and redox imbalance. In contrast, 4-hydroxynonenal (4-HNE) and malondialdehyde (MDA) function not only as terminal products of membrane lipid oxidation but also as bioactive mediators capable of influencing cell proliferation, apoptosis, and intracellular redox regulation, thereby contributing to tumor development (Matsuoka et al., 2025). Experimental evidence further suggests that 4-HNE can modulate the nuclear factor erythroid 2-related factor 2 (Nrf2) antioxidant pathway through oxidative modification of critical cysteine residues within Kelch-like ECH-associated protein 1 (Keap1), facilitating the adaptive survival of stressed or transformed cells under persistent oxidative conditions (Zuo et al., 2019).

**FIGURE 5:**
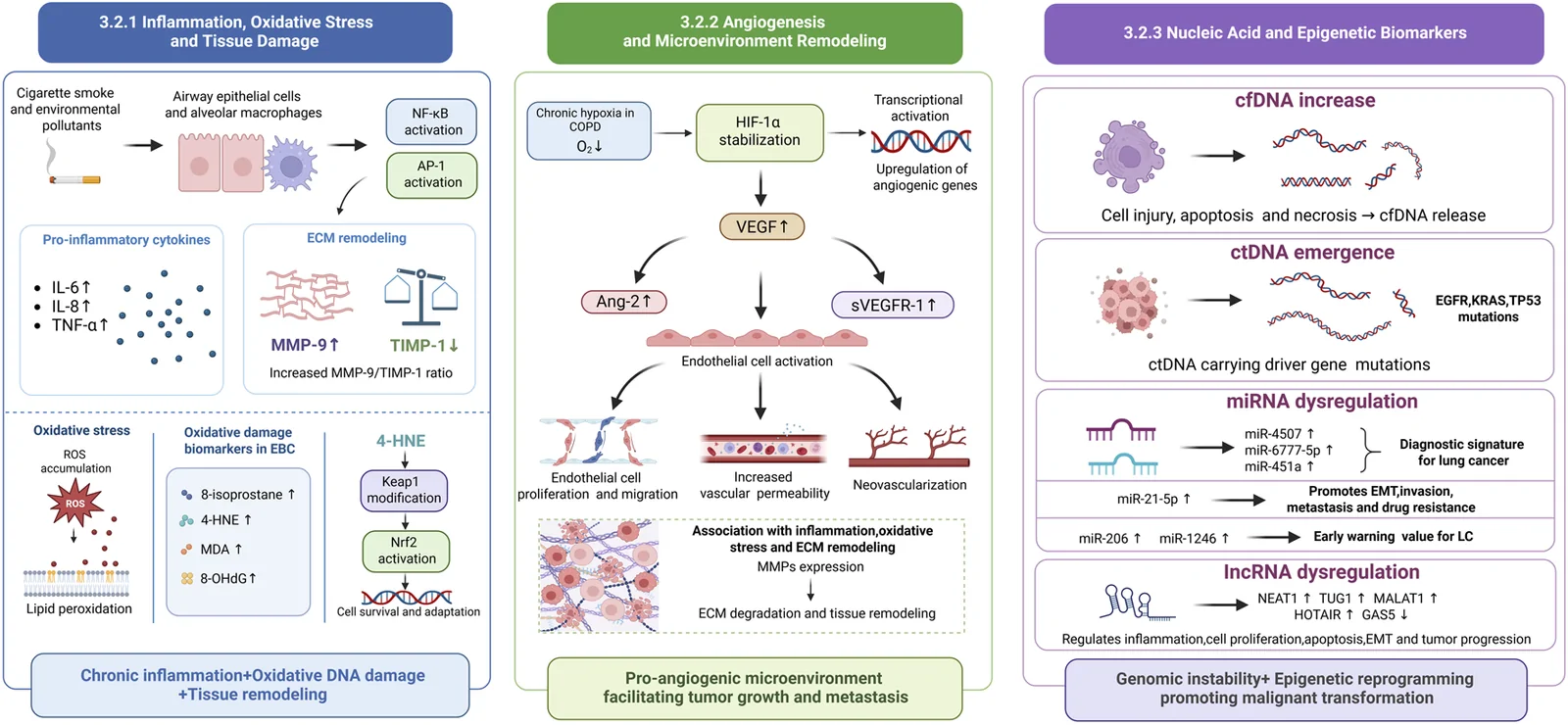
EBC biomarkers and mechanisms of malignant transformation of COPD. Dynamic alterations in EBC biomarkers, including inflammatory mediators, oxidative stress markers, angiogenic factors, and nucleic acids, reflect the transition from chronic airway injury to tumor-promoting microenvironment remodeling. These molecular changes may serve as promising non-invasive biomarkers for the early detection and risk assessment of COPD-related lung carcinogenesis. This figure was created with BioRender.

Ferroptosis, an iron-dependent form of regulated cell death driven by excessive lipid peroxidation, has recently emerged as an important mechanism underlying both COPD pathogenesis and lung carcinogenesis. The lipid peroxidation processes associated with ferroptosis may further promote the accumulation of oxidative products such as 4-HNE and MDA, and may be closely linked to persistent damage to lung tissue and malignant transformation (Chen et al., 2024). Furthermore, studies have found that levels of the oxidative DNA damage marker 8-hydroxydeoxyguanosine (8-OHdG) are persistently elevated in COPD patients several years before they are ultimately diagnosed with lung cancer, suggesting that the accumulation of oxidative damage may precede the onset of clinical malignancy (Córdoba-Lanús et al., 2024) (Figure 5).

However, the clinical specificity of inflammatory and oxidative-stress biomarkers remains limited. Markers such as IL-6, IL-8, TNF-α, H_2_O_2_, 8-isoprostane, and MDA primarily reflect inflammatory or oxidative activity rather than malignant transformation itself (Szucs et al., 2019; Shyam et al., 2021; Barnes, 2020; Taniguchi et al., 2021; Freund et al., 2024; Dos Santos et al., 2022). During acute exacerbations of COPD, intensified airway inflammation and oxidative stress may further increase the levels of these biomarkers, potentially confounding their interpretation as indicators of malignant progression (Antczak et al., 2012). Repeated measurements after clinical stabilization may therefore help distinguish exacerbation-related changes from persistent abnormalities. In addition, combining these markers with more tumor-associated signals, such as characteristic VOC signatures, angiogenesis-related factors, or tumor-derived nucleic acids, may improve the identification of COPD patients at increased risk of lung cancer.

#### 3.2.2 Markers associated with angiogenesis and microenvironmental remodeling

Chronic airflow limitation and structural damage to lung tissue in COPD lead to persistent local hypoxia, which promotes the stable expression of HIF-1α and activates various downstream angiogenesis-related genes; among these, VEGF is one of the most important effector molecules (Wiegman et al., 2020; Zheng and Jin, 2025). Studies have shown that VEGF levels are elevated in EBCs from COPD patients and are further increased in lung cancer patients; these concentration changes are closely associated with enhanced angiogenic activity and an increased risk of lung cancer (Nicola et al., 2021).

VEGF promotes endothelial cell proliferation, migration, and neovascularization while increasing vascular permeability, thereby improving oxygen and nutrient supply to proliferating tumor cells and facilitating metastatic dissemination. In addition to VEGF, angiopoietin-2 (Ang-2) and soluble vascular endothelial growth factor receptor-1 (sVEGFR-1), among other factors associated with vascular remodeling, are also thought to be involved in COPD to lung cancer. By regulating vascular stability, endothelial cell function and local inflammatory responses, these molecules collectively promote the transformation of lung tissue from a state of chronic injury repair to a tumor-promoting angiogenic microenvironment (Qin et al., 2021; He et al., 2024). Angiogenesis is not an isolated event but is closely associated with inflammatory responses, oxidative stress and extracellular matrix remodeling. VEGF-related signaling can further promote the expression of matrix metalloproteinases (MMPs), enhancing extracellular matrix degradation and tissue remodeling, thereby creating favourable conditions for tumor cell invasion and metastasis (Poto et al., 2022) (Figure 5). Consequently, angiogenesis-related factors such as VEGF, Ang-2 and sVEGFR-1 not only reflect the degree of pulmonary hypoxia and vascular remodeling, but may also serve as important candidate EBC biomarkers for assessing the risk of lung cancer transformation in patients with COPD.

#### 3.2.3 Nucleic acid and epigenetic markers

Nucleic acid biomarkers can directly reflect genomic instability and abnormalities in epigenetic regulation, and are considered to be among the most promising EBC biomarkers in the malignant transformation of COPD to lung cancer. In addition to molecules associated with inflammation and oxidative stress, various nucleic acid components can be stably present in EBC, including cfDNA, ctDNA, miRNAs and lncRNAs.

As lung tissue sustains ongoing damage and abnormal cell proliferation, the release of cfDNA and ctDNA gradually increases. COPD patients are in a state of chronic inflammation and oxidative stress over the long term; persistent cellular damage, apoptosis and necrosis can lead to increased cfDNA release. cfDNA levels are closely associated with the degree of inflammation, declining lung function and disease severity. As malignant transformation occurs, some cfDNA originating from abnormally proliferating cells gradually carries tumor-associated genetic alterations and further evolves into detectable ctDNA. Some existing studies have successfully detected ctDNA carrying information on driver gene mutations such as EGFR, KRAS and TP53 in EBC; the results of these tests show a high degree of consistency with tissue biopsies, suggesting that EBC holds promise as an important carrier for non-invasive liquid biopsy in the early stages of lung cancer (Koc et al., 2019; Ryan et al., 2022).

MiRNAs have emerged as one of the most intensively investigated classes of nucleic acid biomarkers in exhaled breath condensate (EBC). Using genome-wide miRNA profiling combined with machine-learning algorithms, a study involving 21 patients with lung cancer and 21 healthy individuals identified a three-miRNA signature consisting of miR-4507, miR-6777–5p, and miR-451a, which demonstrated promising discriminatory performance for lung cancer detection (Pérez-Sánchez et al., 2021). miR-21–5p, a well-characterized oncomiR, is frequently upregulated across multiple malignancies and has been implicated in lung tumor progression through its ability to promote EMT, suppress apoptosis, and enhance cellular invasion and metastatic potential (Khair and Karagöz, 2024). Apart from the EBC study, long-term follow-up studies of some COPD patients have found that circulating miRNAs such as miR-206 and miR-1246 exhibit abnormal expression prior to the onset of lung cancer, suggesting that they may have value as early warning markers for lung cancer (Córdoba-Lanús et al., 2023).

Beyond miRNAs, lncRNAs have emerged as important regulators of chronic airway inflammation, tissue remodeling, and tumor progression in both COPD and lung cancer. Aberrant expression of lncRNAs, including NEAT1, TUG1, MALAT1, HOTAIR, and GAS5, has been documented in pulmonary tissues and is thought to influence multiple biological processes, such as inflammatory signaling, cellular proliferation, apoptosis, and EMT (Sweef et al., 2024). Among these molecules, MALAT1 and HOTAIR are strongly associated with tumor invasion and metastatic dissemination, whereas NEAT1 and TUG1 have been implicated in sustaining chronic inflammatory responses and modulating the tumor-supportive microenvironment during COPD progression and malignant transformation (Figure 5).

## 4 Advances in exhaled breath biomarker detection technology and clinical translation

### 4.1 Analytical technologies for respiratory biomarker detection

The reliability of respiratory biomarkers depends strongly on the analytical platforms used for their detection. For VOC analysis, gas chromatography–mass spectrometry (GC–MS) remains one of the most widely used reference techniques because it enables the separation and molecular identification of individual volatile compounds (Buszewski et al., 2012). Other analytical platforms, including gas chromatography–ion mobility spectrometry (GC–IMS), proton-transfer-reaction mass spectrometry (PTR–MS), and selected-ion flow-tube mass spectrometry (SIFT–MS), offer faster or real-time VOC profiling and may facilitate clinical breath analysis (Smith et al., 2025; Xu et al., 2022; Gasparri et al., 2022; Mortazavi et al., 2025a). However, differences in sampling procedures, compound identification, instrument sensitivity, and data preprocessing can generate substantial inter-study variability, making direct comparison of VOC signatures across cohorts challenging.

For EBC biomarkers, analytical methods vary according to molecular class. Inflammatory cytokines and angiogenic factors are commonly quantified using immunoassays, whereas oxidative-stress-related metabolites can be measured using spectrophotometric, chromatographic, or mass-spectrometry-based approaches (Yoo et al., 2024). Nucleic-acid biomarkers, including miRNAs and tumor-associated DNA alterations, increasingly rely on qRT-PCR, digital PCR, sequencing, and other high-sensitivity molecular platforms (Grätz et al., 2022). Although these approaches have substantially expanded the range of detectable EBC biomarkers, the low and variable concentrations of analytes in EBC remain an important analytical challenge. Differences in condensate collection, dilution, storage, normalization, and assay sensitivity may further contribute to inconsistent findings across studies.

### 4.2 Sensor technologies and AI-driven respiratory omics analysis

Sensor-based platforms offer a faster alternative for respiratory biomarker analysis (Mortazavi et al., 2025b). Nanomaterials such as carbon nanotubes, graphene, and metal–organic frameworks (MOFs) can enhance the detection of low-abundance VOCs and have also been explored for molecular analysis of EBC (Kiejzik et al., 2025; Shao et al., 2025). Rather than identifying individual compounds, electronic-nose (e-nose) systems capture collective sensor responses to VOC mixtures and generate disease-associated breathprints (Dhanush Gowda et al., 2025). Machine learning can further extract informative patterns from these complex signals (Setlhare et al., 2025). More importantly, computational models provide an opportunity to combine breath VOCs with EBC-derived molecules and clinical information, which may be more useful for risk stratification in COPD than relying on a single biomarker (Subawickrama Mallika Widanaarachchige et al., 2025).

However, several technical and methodological limitations currently restrict these applications. E-nose signals can vary with sensor drift, humidity, ambient VOCs, calibration, and sampling procedures (Taha et al., 2025; Dragonieri et al., 2023). For AI-based analysis, relatively small cohorts compared with the large number of measured features increase the likelihood of overfitting and may explain why model performance is not always maintained in independent populations (Gallos et al., 2023). Patient-related factors, including smoking, diet, medication, infection, and comorbidities, add further variability to breath profiles (Dragonieri et al., 2023). Differences in analytical platforms and data processing also make comparisons between studies difficult. Standardized sampling and analytical procedures, together with prospective multicenter validation, are therefore needed before these approaches can be reliably incorporated into COPD surveillance.

## 5 Conclusion

COPD and lung cancer may be viewed as interconnected conditions along a shared pathogenic continuum; pathological processes such as persistent chronic inflammation, oxidative stress, abnormal tissue repair, metabolic reprogramming and pulmonary microecological imbalance collectively drive the progression of lung tissue from chronic injury to malignant transformation. As a key component of respiratory omics, exhaled breath analysis can dynamically reflect changes in the pulmonary microenvironment at the metabolic, inflammatory and molecular levels, providing a new research perspective for identifying early biological events in COPD malignant transformation. Multiple VOCs and EBC biomarkers have shown potential relevance to the biological processes associated with COPD-to-lung cancer progression. For example, aldehydes associated with lipid peroxidation, VOCs linked to abnormalities in glycolysis and amino acid metabolism, as well as inflammatory mediators, products of oxidative damage, angiogenic factors and nucleic acid molecules in EBC, may serve as candidate translational biomarkers linking COPD and lung cancer. These biomarkers not only reflect the biological characteristics of different stages along the disease continuum but also provide a potential basis for the early identification and dynamic monitoring of high-risk populations for lung cancer. Future research should focus on establishing longitudinal follow-up cohorts, with particular attention to the dynamic changes in biomarkers during the progression from COPD to lung cancer, and promote multi-omics integrated analysis of exhaled VOCs, EBC molecular biomarkers, imaging and clinical characteristics. At the same time, by combining electronic noses, nanobiosensors and AI-assisted risk prediction models, a precision early-warning system suitable for high-risk COPD populations should be established, providing new strategies and directions for the precise prevention and control of respiratory cancers.

## Glossary

4-HNE: 4-hydroxynonenal
8-isoprostane: 8-isoprostaglandin F_2_α
AhR: aryl hydrocarbon receptor
ALDH: aldehyde dehydrogenase
ALK: anaplastic lymphoma kinase
Ang-2: angiopoietin-2
AP-1: activator protein-1
ARE: antioxidant response element
ATP: adenosine triphosphate
BH4: tetrahydrobiopterin
CBS: cystathionine β-synthase
CEA: carcinoembryonic antigen
cfDNA: cell-free DNA
COX-2: cyclooxygenase-2
COPD: chronic obstructive pulmonary disease
CSE: cystathionine γ-lyase
ctDNA: circulating tumor DNA
DDR: DNA damage response
DMS: dimethyl sulphide
EBC: exhaled breath condensate
EGFR: epidermal growth factor receptor
E-nose: electronic nose
EMT: epithelial–mesenchymal transition
GC–MS: gas chromatography–mass spectrometry
GC–IMS: gas chromatography–ion mobility spectrometry
GSH: glutathione
GST: glutathione S-transferase
GLUT1: glucose transporter 1
GPX: glutathione peroxidase
GPX4: glutathione peroxidase 4
H_2_O_2_: hydrogen peroxide
HIF-1α: hypoxia-inducible factor-1α
HK2: hexokinase 2
HO-1: heme oxygenase-1
IDO1: indoleamine 2,3-dioxygenase 1
IL: interleukin
JAK: (Janus kinase)
KRAS: Kirsten rat sarcoma viral oncogene homolog
Kyn: kynurenine
LC: lung cancer
LDCT: low-dose computed tomography
LDHA: lactate dehydrogenase A
lncRNA: long non-coding RNA
LPS: lipopolysaccharide
LTB4: leukotriene B4
MDA: malondialdehyde
miRNA: microRNA
MMP: matrix metalloproteinase
mtDNA: mitochondrial DNA
MYC: MYC proto-oncogene
MyD88: myeloid differentiation primary response 88
NADPH: nicotinamide adenine dinucleotide phosphate, reduced form
NF-κB: nuclear factor kappa B
NQO1: NAD(P)H quinone dehydrogenase 1
Nrf2: nuclear factor erythroid 2-related factor 2
PAH: phenylalanine hydroxylase
PAMPs: pathogen-associated molecular patterns
PFK: phosphofructokinase
PGE2: prostaglandin E2
PI3K: phosphoinositide 3-kinase
PPP: pentose phosphate pathway
PTR–MS: proton-transfer-reaction mass spectrometry
PUFAs: polyunsaturated fatty acids
RNS: reactive nitrogen species
ROS: reactive oxygen species
SASP: senescence-associated secretory phenotype
SIFT–MS: selected-ion flow-tube mass spectrometry
STAT3: signal transducer and activator of transcription 3
sVEGFR-1: soluble vascular endothelial growth factor receptor-1
TDO: tryptophan 2,3-dioxygenase
TGF-β: transforming growth factor-β
TIGAR: TP53-induced glycolysis and apoptosis regulator
TIMP-1: tissue inhibitor of metalloproteinases-1
TLR4: Toll-like receptor 4
TNF-α: tumor necrosis factor-α
TP53: tumor protein p53
Trp: tryptophan
Trp–Kyn: tryptophan–kynurenine
VEGF: vascular endothelial growth factor
VOCs: volatile organic compounds
Wnt: Wingless/Integrated signaling pathway
